# Victimization of the Substance Abuse and Sexual Behaviors among Junior High School Students in Cambodia

**Published:** 2018-03

**Authors:** Yat YEN, Yumin SHI, Bunly SOEUNG, Rathny SUY, Muhammad Tayyab SOHAIL

**Affiliations:** 1. School of Public Affairs, University of Science and Technology of China, Hefei, China; 2. Dept. of Social Work, National Institute of Social Affairs, Phnom Penh, Cambodia; 3. Faculty of Social Sciences, Svay Rieng University, Svay Rieng, Cambodia; 4. School of Public Administration, China University of Geosciences (Wuhan), Wuhan, Hubei, China

**Keywords:** Substance abuse, Sexual behaviors, Victimization, Cambodia

## Abstract

**Background:**

We examined the current prevalence of substance abuse and sexual behaviors among junior high school students and the relationships between substance abuse, sexual behaviors, and victimization using multiple mediations.

**Methods:**

Overall, 1703 junior high school students from the 2013 Cambodia Global School-based Health Survey were selected for the study. The descriptive statistics were performed in IBM SPSS to determine the prevalence of substance abuse, sexual behaviors, and victimization. The Process Macro was installed in Regression of the SPSS to test the hypotheses and mediations.

**Results:**

The majority of students who used alcohol (15.4%), drugs (3.05%), and had sexual intercourse (12.45%), were male aged 14–15, and in grade 7. These students were very vulnerable to many risky behaviors, including bullying (22.20%), physical attacks (20.96%) and fights (14.50%), unintentional accidents (21.32%), and suicidal attempts (5.05%). All three hypotheses were significantly supported. Of the potential mediators examined, drug use is the most important mediator.

**Conclusion:**

The substance abuse and reproductive health are national problems, but abusive behaviors among students are of particular concern. Explicit policies and awareness programs of such problems at the high school level need to be made and called for public participation, particularly the school authorities and parents.

## Introduction

Alcohol use among adolescents is a critical public concern worldwide (1). The negative influences of substance abuse on mental and physical health were found such as being a victim of sexual assault (2, 3), drug use (4, 5), smoking cigarettes (6, 7), suicidal attempts (8), bullying (9, 10), physical violence or attacks (10, 11), various kinds of unintentional injuries (12, 13) and road crashes (13, 14). A strong relationship between alcohol use and drug use was found as well as the relationships between both alcohol and drugs and sexual behaviors (15, 16).

The US National Center for Addiction and Substance Abuse reported that on campuses 95%, 90%, and 80% of violent crime, rape, and vandalism were alcohol-related respectively. About 35%–70% of college students engaged in sexual activity because of alcohol use while 60% of college women diagnosed with a sexually transmitted disease was drunk at the time of infection. Totally, 240000 to 360000 of the nation’s 12 million undergraduates would ultimately die from alcohol-related problems which were more than a number of master and Ph.D. students combined (17).

The adolescents are more vulnerable to substance abuse and sexual assault than other groups of the population. Approximately 50% of all rape victims were adolescents of whom females consistently exceed those of males (16). Substance abuse places adolescents at increased vulnerability to sexual assault due to the physiological effects of substances and risky situations that motivate perpetrators (18). In addition, the adolescents who have assault histories are more likely to be re-victimized in the future (19). Some factors of victimization have been identified, such as substance abuse (3, 18), low self-esteem (20), depression (21), eating disorders (22), high-risk sexual practices (e.g., without using a condom) (23), being attacked (24), and family background (25).

There is very scant information about the victimization of substance abuse among Cambodian students, particularly junior high school students (JHSs). In 2013, a very first study of Cambodia Global School-based Student Health Survey (C-GSHS) among students from grades 7–12 was done but focused on many health-related behaviors (26). In 2014, another study of people’s perceptions of alcohol use was conducted by the Royal University of Phnom Penh and Cambodian Movement for Health (27). None of these two studies focused specifically on the victimization of JHSs resulting from alcohol use, drug use, and sexual behaviors. Moreover, a critical social phenomenon of substance abuse that is a leading cause of fatalities among adults recently is traffic crashes. In 2013, there were 4353 road crashes which caused 16227 casualties, of which 1950 were fatalities and 5349 were seriously injured (on average of 5 fatalities and 15 serious injuries per day). The human factors contributed 95% to crashes, and drunk driving stood as the second leading factor. The vulnerable groups were farmers (39%), workers (19%), and students (13%), whose median age was 20–24 yr old and males represented 80% of fatalities (28). An increase in illicit drug use among adults is also a current social concern. In 2015, the police seized 3061 cases of illicit drugs, with an increase of 128.94% compared to 1337 in 2014, and arrested 7008 suspects, with an increase of 123.04% compared to 3142 in 2014 (29).

**Fig. 1:**
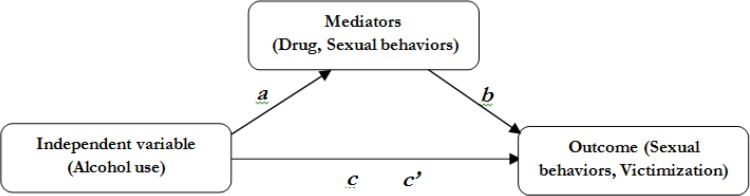
The conceptual models of mediated pathways of AL to SB and Vict

To get more insight of the substance abuse and sexual behaviors among JHSs in Cambodia, this study, aimed to examine the prevalence of substance abuse and sexual behaviors among JHSs; to find the relationships among substance abuse, sexual behaviors, and victimization by using multiple mediators. Three hypotheses have been formed as follows (Fig. 1):

H1. The relationship between alcohol use (AL) and sexual behaviors (SB) is significantly mediated by drug use (Drug).
H2: The relationship between alcohol use (AL) and victimization (Vict) is significantly mediated by drug use (Drug).
H3: The association between alcohol use (AL) and victimization (Vict) is significantly mediated by sexual behaviors (SB).

The a-path is an effect of AL on Drug and SB; b-path is an effect of Drug on SB and Vict; c-path is a direct effect of AL on SB and Vict, and c’-path is an indirect effect of AL on SB and Vict. The c’-path shall be reduced and smaller than c-path when it is mediated.

## Materials and Methods

### Study design and sample

The study utilized the data of the 2013 C-GSHS (http://www.cdc.gov/gshs) which was a primary survey of students from grades 7–12 whose ages were 11–18.

The C-GSHS was initiated by the WHO with technical assistance from the US Centers for Disease Control and Prevention (CDC) (30). It was a two-stage cluster sample design, which aimed to produce data representing of all students in grades 7–12 throughout the country during 2012–2013. Firstly, 25 schools in urban and 25 schools in rural were selected, and secondly, classes were randomly selected, and all students in these classes were qualified to participate. The survey composed of 69 questions that measured alcohol use, dietary behaviors, drug use, mental health, physical activity, sexual behaviors, tobacco, violence and unintentional injury. In order to achieve the purpose of the study, only 22 questions were used to measure alcohol, drugs, sexual behaviors, and victimization meanwhile only students in grade 7–9 were kept so that students in grade 10–12 were excluded. A reason to which authors excluded students in grade 10–12 was that the authors wanted to clarify the vital factors of vulnerable victimization among the JHSs specifically.

### Data cleaning

Originally, there were 3788 students from grade 7–12, but when students in grade 10–12 were excluded, there were only 2164 (57.13%) students left. There were two stages of data cleaning. Firstly, a number of students of grade 10–12 and other irrelevant variables were discarded from the original data using IBM SPSS Modeler v.17.0. Secondly, all missing values were also discarded, and finally, 461 students (21.30% of 2164) were removed, and only 1703 students (78.70%) and 22 questions remained.

### Measurements

The questionnaire consisted of three parts: 1) demographic information: age, gender, and grade; 2) alcohol, drug, and sexual behaviors; and 3) victimization. To measure each part, the students were asked to choose an answer from the available options. For example, in part two, students can choose *“No=1 or Yes=2”* whether or not they had ever drunk alcohol previously. Likely, the age at first alcohol use was measured by “*never drank alcohol (1)* to *≥18 yr old (5)”*. A number of drinks that students drank in the past 30 d were scored by *“never drank alcohol (1)* to *≥ 5 drinks (5)”.* Frequencies of being drunk were measured from “*0 times (1)* to *≥10 times (4)”.* To measure the experience of drug use by giving *“No=1 or Yes=2”* options while the first age of drug use was also explored. The options of “*never use (1) and 20 times (5)”* were available for the frequencies of using marijuana and amphetamines in a lifetime. About the sexual behaviors, students could choose *“No=1* or *Yes=2”* whether or not they had ever had sex, whereby a number of sex partners were available from *“never had sex (1)* to *5 or more partners (5)”*. They were also elicited to answer whether or not they used the condom/birth control whenever they had sex by offering, *“never had sex (1), No (2), and Yes (3)”*. Lastly, the victimization of bullying, physical attacks, fights, injuries, and suicide attempts resulting from alcohol, sexual behaviors, and drugs was investigated using “*No=1 or Yes=2”* optional answers.

### Ethics approval

The 2013 Cambodia GSHS questionnaire was designed to protect respondents’ rights by allowing for anonymous and voluntary participation. The procedures and questionnaire were also administered and approved by a group of experts who were surveying supervisors and administrators from Ministry of Health and Ministry of Education, Youth and Sports, Cambodia. An official permission and consent of school principals and classroom teachers were requested and obtained from both ministries and selected schools. The students completed a self-administered cross-sectional questionnaire during one classroom period.

### Statistical analysis

The IBM SPSS v.20.0 (IBM, Armonk, NY, USA) was employed to find the prevalence of alcohol use, sexual behaviors, drug use, and victimization. The Cronbach’s alpha (α) was used to examine the reliability and validity of the data. The α value of ≥0.70 is acceptable for social sciences(31). The multiple mediators were executed based on criteria (32–34), that an independent variable *(X)* must have a significant effect on a mediator *(M),* and *X* must have a significant effect on a dependent variable *(Y)* in a regression model without adjustment for M. Subsequently, *M* must have a significant effect on Y, and the effect of *X* on *Y* decreases when it is adjusted for *M* (35). To measure the strength of mediations, researchers should take into account the effect proportion mediated (ratio *P_M_*) which is a computation of the ratio of the indirect effect to the total effect of X→Y. The higher value of ratio *P_M_* is the higher effect of X→Y (34). The P_M_ is computed by ${\text{P}}_{\text{M}}=\frac{\text{a}*\text{b}}{\text{a}*\text{b}*\text{c}\prime }\times 100$ , where *a* is an effect of X → M, *b* is an effect of M→Y, and *c*’ is an indirect effect of X→Y. When the indirect effect (a*b) equals the total effect (c-path), then it shows that the effect of X→Y is completely mediated by M. In such case, there is no direct effect of X→Y and c’-path is equal to zero. On the other hand, when the indirect effect does not equal the total effect but it is smaller, and of the same sign, it shows that the effect of X→Y is partially mediated by M (34, 35). In this study, three models of multiple mediations had been performed continuously. In the first model (H_1_), the SB (dependent variable) was predicted by AL (independent variable) and mediated by Drug (mediator). Therefore, path coefficients of AL must have a significant effect on Drug (a-path) and SB (c-path). At the same time, Drug must have a significant effect on SB (b-path). The effect of AL on SB (c’-path) was decreased when it was mediated by Drug. In the second model (H_2_), the prediction of AL on Vict was mediated by Drug. Lastly, in the third model (H_3_), Vict was predicted by AL and mediated by SB. The multiple mediations of the study were analyzed in The Process Macro v.2.16 (33, 35, 36) to find the relationships among the variables. The bootstrap 5000 samples were selected and calculated in the Process Macro to find indirect effects, estimates and 95% of confidence intervals (BC and BCa) (36). To do this, firstly, Process Macro (http://processmacro.org) was installed onto Regression of IBM SPSS. Lastly, Heteroscedasticity-consistent SEs, OLS/ML confidence intervals, Effect size, Sobel test and Total effect model were applied. The *P-*values is significant at *P≤0.05*.

## Results

The value of Cronbach’s alpha is 0.728, which is higher than a common threshold (0.70) of reliability and validity of all variables. Thus, the data of this study are valid and reliable. Table 1 shows that female students accounted for 55.7% among which 41.8%, 31.5%, and 26.7% were students in grade 7^th^, 8^th^, and 9^th^ respectively. The majority of the students aged 13–15 (N=1292, 75.90%, M=14.46, Md=14.00, SD=1.344).

**Table 1:** Demographic Characteristics of the Respondents

***Variables***		***Gender***		***N (%)***	***M***	***Md***	***SD***
		**Male (%)**	**Female (%)**				
		755 (44.3)	948 (55.7)	1703 (100)	1.56	2.00	.497
Grade	7	319 (44.7)	393 (55.3)	712 (41.8)	1.85	8.00	.814
	8	236 (43.9)	301 (56.1)	537 (31.5)			
	9	200 (44.1)	254 (55.9)	454 (26.7)			
Age (yr)	≤11	2 (33.3)	4 (66.7)	6 (0.4)	14.46	14.00	1.344
	12	12 (19.0)	51 (81.0)	63 (3.7)			
	13	153 (42.7)	204 (57.3)	357 (21.0)			
	14	225 (43.5)	292 (56.5)	517 (30.4)			
	15	185 (44.3)	233 (55.7)	418 (24.5)			
	16	98 (46.0)	115 (54.0)	213 (12.5)			
	17	50 (61.7)	31 (38.3)	81 (4.8)			
	≥18	30 (62.5)	18 (37.5)	48 (2.8)			

Note: N= total samples, %=percentage of male or female students for each grade and age category, *Mean= mean value, Md=median, SD=standard deviation*

Overall, 271 (15.4%, male=170 vs. 101, M=1.14, SD=.349) students whose age was 15 and in grade 7 reported drinking alcohol at a higher proportion than other age groups (Table 2).

**Table 2:** Proportions of AL, SB, Drug, and Vict categorized by age, grade, and gender

***Variables***		***Age(yr)***								***Grade***			***Gender***		***N(%)***	***Md***	***M***	***SD***
		**≤11**	**12**	**13**	**14**	**15**	**16**	**17**	**≥18**	**7**	**8**	**9**	**M**	**F**				
Had alcohol	No	5	58	318	449	341	170	61	30	619	449	364	585	847	1432 (84.09)	1.00	1.14	.349
	Yes	1	5	39	68	77	43	20	18	93	88	90	170	101	271 (15.91)			
Had ever used drugs	No	5	63	350	499	404	204	80	46	689	515	447	727	924	1651 (96.95)	1.00	1.11	.318
	Yes	1	0	7	18	14	9	1	2	23	22	7	28	24	52 (3.05)			
Ever had sex	No	5	58	322	455	364	177	73	37	623	463	405	639	852	1491 (87.55)	1.00	1.02	.155
	Yes	1	5	35	62	54	36	8	11	89	74	49	116	96	212 (12.45)			
Victim of bullying in past 30 d	No	4	52	281	398	325	170	58	37	548	419	358	580	745	1325 (77.80)	1.00	1.21	.406
	Yes	2	11	76	119	93	43	23	11	164	118	96	175	203	378 (22.20)			
Victim of attacks in past 12 months.	No	4	51	277	407	341	169	59	38	541	436	369	564	782	1346 (79.04)	1.00	1.19	.396
	Yes	2	12	80	110	77	44	22	10	171	101	85	191	166	357 (20.96)			
Victim of fights in past 12 months	No	5	55	297	447	362	183	62	44	584	475	396	612	843	1455 (85.44)	1.00	1.14	.345
	Yes	1	8	60	70	56	30	19	4	128	62	58	143	105	248 (14.56)			
Victim of injuries in past 12 months	No	6	54	295	412	323	158	57	35	565	406	369	578	762	1340 (78.68)	1.00	1.20	.401
	Yes	0	9	62	105	95	55	24	13	147	131	85	177	186	363 (21.32)			
Victim of suicide attempt in past 12months	No	6	63	344	488	399	193	74	44	675	511	425	721	890	1611 (94.60)	1.00	1.05	.214
	Yes	0	0	12	25	18	20	7	4	32	25	29	33	53	86 (5.05)			

**Note**: Md=median, M=mean, SD=standard deviation. N (%) = a total response that respondents answered “yes/no” to each question

In addition, 89 out of 271 alcohol users reported being drunk at least one time during the past 30 d. Furthermore, 212 (12.45%, male=116 vs.96, M=1.11, SD=.318) students whose age 14 and in grade 7 reported ever having sexual intercourse, of whom 19 had more than 2 sex partners, and only a few of them used the condom or birth control at their last sexual intercourse. Another 52 (3.05%, M=1.02, SD=.155) students, who were 14 yr old and in grade 8, ever used drugs and at least 29 students used Marijuana and other 17 used Amphetamines.

Many students had been victimized by bullying, attacks, fights, injuries, and suicide attempts. Of the total, 378 students who were 14-yr-old females and in grade 7 were bullied more often than those male students in the last 30 d. In contrast, 191 out of 357 male students, whose age was 14 and in grade 7, experienced physical attacks and involved with fights more frequently than those female students in the past 12 months. Among 248 students victimized by physical fights, 143 were male, aged 14 and in grade 7. Reversely, in the past 12 months, 186 out of 363 were female students were injured and of the total, 86 students had attempted to commit suicide, and 53 were females.

All three models of the study were significantly supported (Table 3). The Model 1 exhibited that AL had a significant effect on SB (β=.073, t=3.975, *P*<.0001) and was significantly mediated by Drug (β=.086, t=5.882, *P*<.0001) while the Drug also had a significant effect on SB (β=.271, t=3.799, *P*<.0001). Model 2 showed that AL had also significantly influenced on Vict (β=.140, t=9.724, *P*<.0001) and was statistically mediated by Drug (β=.086, t=5.882, *P*<.0001) while Drug also had a significant effect on Vict (β=.0198, t=3.687, *P*<.0002). Lastly, the Model 3 was satisfactorily accepted. The AL was strongly correlated with Vict (β=.143, t=9.979, *P*<.0001) and was significantly mediated by SB (β=.097, t=5.069, *P*<.0001) while SB was also correlated with Vict (β=.143, t=2.922, *P*<.0003).

**Table 3:** Multiple mediators of effects of AL on SB and Vict through Drug

***Hypothesis.***	***Correlation.***	***Coefficients.***	***s.e***	***t***	***P***	***Est.***	***Sobel Z***	***Bootstrapping***		***P_M_***	***Support.***
								**BC95% CI**	**BCa95% CI**		
(H_1_)	AL→Drug	0.086	0.015	5.882	0.0000	0.023	3.160	0.011–0.040	0.031–0.104	24.10	**Yes**
	Drug→SB	0.271	0.071	3.799	0.0001						
	AL_ind._→SB	0.073	0.019	3.975	0.0001						
	AL_dir._→SB	0.097	0.019	5.069	0.0000						
(H_2_)	AL→Drug	0.086	0.015	5.882	0.0000	0.017	3.092	0.008–0.030	0.017–0.060	10.80	**Yes**
	Drug→Vict	0.198	0.054	3.687	0.0002						
	AL_ind._→Vict	0.140	0.014	9.724	0.0000						
	AL_dir._→Vict	0.157	0.014	10.912	0.0000						
(H_3_)	AL→SB	0.097	0.019	5.069	0.0000	0.014	2.495	0.006–0.023	0.013–0.048	8.80	**Yes**
	SB→Vict	0.143	0.049	2.922	0.0003						
	AL_ind._→Vict	0.143	0.014	9.979	0.0000						
	AL_dir._→Vict	0.157	0.014	10.912	0.0000						

**Note:** P_M_ is a ratio of indirect effect to total effects of X→Y. s.e=Standard deviation Error; Est=Estimated effect that is deducted from direct effect by indirect effect; Dir. and Ind.= Direct and indirect effects; t= t-value; *P*=*P*-value (*.*P*<.05; **.*P*<.01; ***. *P*<.001); BC=bias corrected; BCa=bias corrected accelerated; 5000 bootstrap samples

**Fig. 2:**
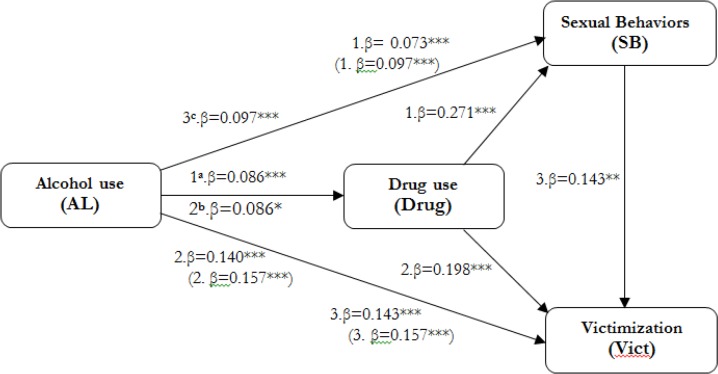
Mediations illustration: ^a, b^AL predicted SB and Vict indirectly mediated by Drug; ^c^AL predicted Vict indirectly mediated by SB. **. *P*<.01; ***, *P*<.001

Fig. 2 depicted the results that were consistent with the criteria of Baron and Kenny (1986) who explained that a path coefficient of X→Y would be reduced when it was mediated by mediator M (32). In the Model 1, a path coefficient of AL on SB was significantly reduced when it was accounting for by Drug (β= 0.097, β= 0.073, *P*<.0001). Model 1 showed that approximately 24.10% of the variance in the SB could be accounted for by *P_M_* of AL, which was the highest among other ratios. Likely, a path coefficient of AL in the Model 2 on Vict was significantly downgraded when it was mediated by Drug (β= 0.157, β=0.140, *P*<.0001) with about 10.8% of the variance in Vict could be accounted for by *P_M_* of AL. Lastly, a path coefficient in Model 3 of AL to Vict was statistically decreased through SB (β= 0.157, β=0.143, *P*<.0001) with about 8.80% of the variance in Vict could be explained by *P_M_* of AL (32).

The estimated indirect effect in the Model 1 was 0.023 while in Model 2 and 3 were 0.017 and 0.014 respectively. Drug partially mediated the relationships between AL and SB and between AL and Vict whereas SB mediated the relationship between AL and Vict. Of the potential mediators examined, the mediator (Drug) of the relationship between AL and SB is likely the most important.

## Discussion

The victimizations of alcohol use and unsafe sexual behaviors among adolescents remain a key social and public health problem. The JHSs in Cambodia were very vulnerable to the risks resulting from this dilemma. The misperception of being drunk at the first sexual intercourse is a common reason of sexual experience (37) that would lead adolescents to drink at a very young age and experience unsafe sexual behaviors (38). The findings of this study were consistent with previous studies on alcohol use, sexual behaviors, and victimization. For instance, there were 89 out of 271 JHSs reporting ever being drunk at least one time during the past 30 d whereby about 26 JHSs drank more than 4 drinks in the past 30 d, so-called binge drinkers according to College of Alcohol Study at Harvard School of Public Health (39).

Surprisingly, many JHSs, who were just 14 and in grade 7, reported drinking alcohol, using drugs, and having sex. They also faced with higher risks compared with other JHSs in grades 8–9. Although the mean age of first sexual behaviors among adolescents varies from one country to another, the Cambodian JHSs aged just 13–15 that were too young to experience such risky relationships. To prevent such risky behaviors, parents, school authorities, and other stakeholders shall pay more attention to this group. Comparing with other developing countries in Africa, the median age at the first sex ranges from 15.5 and 18.5 yr old among women in most Sub-Saharan African countries (38, 40). Sexual experience was very common among Ugandan female adolescents than those males (69% vs. 55%) where the mean age was 17.1 for females and 17.0 for males. The factors associated with initiation of sexual experience were thought to be alcohol use, older age, non-enrollment in school, and being orphan among men (38). The mean age of first sex was 15.7 yr old for Ethiopian students, and one-fourth of 1031 students had multiple sexual partners (41). Looking at Australia, the median age at first sex was 14 (37) while in the United States, aged 25–39 for men and aged 20–29 for women (42). What a real concern is that the young age at first sex is highly reported of unwanted pregnancy (43), a number of lifetime sexual partners (37), and other sexual diseases such as HIV and STDs (41, 44–46). Overall, 358 out of 1065 Ghanaian students reported ever being forced to have sexual intercourse when they did not want to (23). Girls who were less than 14 yr old were more likely not to use contraception or take a long time before they started using it; however, they were more likely to have several sex partners (47). Notably, 82% of sexually active high school female students aged 14–19 in Australia had ever been pregnant, 36% of whom terminated their first pregnancy and 55% continuing, and 9% reporting multiple pregnancies (37). The relationships of AL, Drug, SB, and Vict were significantly and consistently denoted and proven. Realistically, any involvement in AL and Drug results in risky consequences for both individual users and secondhand effects (48). Our study showed that the direct effect of each model was significantly reduced when it was accounted for by mediators. The indirect effect from AL to SB in the Model 1 does not equal the total effect but is smaller and of the same sign. In this case, the direct effect of AL on SB is partially mediated by the Drug. It is apparently the same for Model 2 that the indirect effect from AL to Vict does not equal the total effect but is smaller and of the same sign. Therefore, the direct effect of AL on Vict is also partially mediated by Drug. In the Model 3, the indirect effect from AL to Vict also does not equal the total effect so that the direct effect of AL to Vict is partially mediated by SB as well.

Our study indicated that Drug was a potential mediator between AL and SB as well as between AL and Vict. The drug, in addition to its acute effects, serves as a general factor for SB and Vict. The adolescents, who use alcohol, of course facing many problems, would face higher risks when they are addicted to another substance such as drugs.

Due to the scope and nature of the study were mainly focused on only junior JHSs, not represented other groups of students; therefore, the further studies are needed to get more in-depth findings. Firstly, further studies to investigate senior high school students to find how it is different between these groups of students in order to take effective measures to prevent and reduce risks of substance abuse and unsafe sexual behaviors. Secondly, another study to investigate the adults who are the same age as the students, but they have not enrolled or dropped out school because this group of adolescents and adults is more vulnerable to many risky behaviors due to knowledge and free time availability.

## Conclusion

The Cambodian JHSs were more likely to use alcohol and drugs at an early age, while some others even involved sexual intercourse. The students who involved with such risk behaviors were commonly facing many dangerous consequences, including bullying, physical attacks and fights, unintentional accidents, and suicide attempts. Therefore, it is imperative for stakeholders, including parents, clinicians caring for adolescents, school authorities, and development partners to collaborate with the government to educate and prevent students from involving with substance use and risk behaviors.

## Ethical considerations

Ethical issues (Including plagiarism, informed consent, misconduct, data fabrication and/or falsification, double publication and/or submission, redundancy, etc.) have been completely observed by the authors.
